# *HvCKX2* gene silencing by biolistic or *Agrobacterium*-mediated transformation in barley leads to different phenotypes

**DOI:** 10.1186/1471-2229-12-206

**Published:** 2012-11-07

**Authors:** Wojciech Zalewski, Wacław Orczyk, Sebastian Gasparis, Anna Nadolska-Orczyk

**Affiliations:** 1 Plant Breeding and Acclimatization Institute - National Research Institute, Radzikow, 05-870, Błonie, Poland

**Keywords:** RNAi silencing, *HvCKX2*, Barley, Genetic transformation, *Agrobacterium*, Microprojectile bombardment

## Abstract

**Background:**

*CKX* genes encode cytokinin dehydrogenase enzymes (CKX), which metabolize cytokinins in plants and influence developmental processes. The genes are expressed in different tissues and organs during development; however, their exact role in barley is poorly understood. It has already been proven that RNA interference (RNAi)-based silencing of *HvCKX1* decreased the CKX level, especially in those organs which showed the highest expression, i.e. developing kernels and roots, leading to higher plant productivity and higher mass of the roots [1]. The same type of RNAi construct was applied to silence *HvCKX2* and analyze the function of the gene. Two cultivars of barley were transformed with the same silencing and selection cassettes by two different methods: biolistic and via *Agrobacterium*.

**Results:**

The mean *Agrobacterium*-mediated transformation efficiency of Golden Promise was 3.47% (±2.82). The transcript level of *HvCKX2* in segregating progeny of T_1_ lines was decreased to 34%. The reduction of the transcript in *Agrobacterium*-derived plants resulted in decreased CKX activity in the developing and developed leaves as well as in 7 DAP (days after pollination) spikes. The final phenotypic effect was increased productivity of T_0_ plants and T_1_ lines. Higher productivity was the result of the higher number of seeds and higher grain yield. It was also correlated with the higher 1000 grain weight, increased (by 7.5%) height of the plants and higher (from 0.5 to 2) numbers of spikes.

The transformation efficiency of Golden Promise after biolistic transformation was more than twice as low compared to *Agrobacterium*. The transcript level in segregating progeny of T_1_ lines was decreased to 24%. Otherwise, the enzyme activity found in the leaves of the lines after biolistic transformation, especially in cv. Golden Promise, was very high, exceeding the relative level of the control lines. These unbalanced ratios of the transcript level and the activity of the CKX enzyme negatively affected kernel germination or anther development and as a consequence setting the seeds. The final phenotypic effect was the decreased productivity of T_0_ plants and T_1_ lines obtained via the biolistic silencing of *HvCKX2*.

**Conclusion:**

The phenotypic result, which was higher productivity of silenced lines obtained via *Agrobacterium*, confirms the hypothesis that spatial and temporal differences in expression contributed to functional differentiation. The applicability of *Agrobacterium*-mediated transformation for gene silencing of developmentally regulated genes, like *HvCKX2*, was proven. Otherwise low productivity and disturbances in plant development of biolistic-silenced lines documented the unsuitability of the method. The possible reasons are discussed.

## Background

*CKX* genes belong to a small family of genes coding cytokinin dehydrogenase, which metabolizes cytokinins in plants. In *Arabidopsis* the genes differ for tissue and organ-specific expression [2] and CKX enzymes showed various biochemical properties and subcellular localization [2,3]. Spatial and temporal differences in expression may contribute to functional differentiation [1,4]. The expression of *HvCKX1* was highest in the roots and developing spikes of barley. The transcript reduction in these organs (via RNA-mediated gene silencing) resulted in higher plant productivity and mass of the roots [1]. The role of the other *HvCKX* genes in barley is poorly understood. Two of them, *HvCKX2* and *HvCKX3,* were cloned and the expression of one caused a cytokinin-deficient phenotype in the heterologous host plants of tobacco [5]. Full-length *OsCKX2* homologues of barley *HvCKX2.1* and *HvCKX2.2* were characterized with a comparative analysis [6].

Barley is fourth among important cereal species with respect to worldwide production. It has also been suggested as a valuable diploid cereal model in genetic studies as well as in Poaceae biology [7,8]. The newly developed biotechnological tool of gene silencing by means of RNA interference makes it possible to knock out or essentially decrease the expression of selected native genes to analyze their function. The efficiency of gene silencing essentially increased with the application of self-complementary “hairpin” hpRNA cassettes [9-11]. For this purpose silencing cassettes containing fragments of the gene of interest in sense and antisense orientation separated by an intron are introduced into the plant genome by genetic transformation. During transcription, a double-stranded hairpin RNA (hpRNA) is formed and recognized by the plant machinery to induce a silencing signal, which is short interfering RNA (siRNA). The homology of siRNA to any transcript, for example the mRNA of the gene, initiates the degradation process and posttranscriptional gene silencing (PTGS). Nowadays, this is the best way of obtaining the “mutant”/changed phenotype for analysis of gene function and plant improvement, especially for species with large genomes, like cereals, for which real mutants are for most of the genes unavailable [1,4,12-15].

There are two basic methods of cereal transformation: indirect and direct. The indirect method is based on the introduction of the plasmid-carrying gene construct/silencing cassette to the plant cell by means of *Agrobacterium*. The direct method uses microprojectile bombardment (biolistic or particle bombardment method). The *Agrobacterium*-mediated method is recommended as the method of choice, because of the introduction, in most cases, of one copy of a non-rearranged or slightly rearranged transgene. However, the efficiency of the method for monocots is still unsatisfactory. The biolistic method was the first one developed and applied in cereals. However, after years of research the disadvantages of the method seemed to be more limiting than that mediated via *Agrobacterium*[16,17]. The most distinctive is the presence of multiple copies of the introduced gene with large rearrangements. An epigenetic consequence of the physically and genetically destructive biolistic method might be the somaclonal variation, which reflects the adaptation process of cells to a different environment [18]. Krizova et al. [19] suggest that epigenetic changes associated with dedifferentiation might influence regulatory pathways mediated by the trans-PTGS processes. Both pathways of silencing by PTGS and transcriptional gene silencing (TGS) might be influenced by environmental and developmental factors [20]. All the unprofitable effects influence transgene cassette expression, which is the main problem in the application of genetic transformation.

The advantages and disadvantages of both indirect and direct methods have already been evaluated in many species, including the model for cereals, rice [21] and barley [22,23]. In most of the papers the comparison of both methods was based on the expression cassette containing some marker or reporter transgenes. We compared the silencing effect of one native gene of barley, *HvCKX2*, directed by the same silencing cassette but introduced by the *Agrobacterium*-mediated and biolistic method. In both methods the same selection cassette was used as well.

We found that the method of genetic transformation used for the silencing of developmentally regulated genes strongly influenced the plant phenotype. The advantages of *Agrobacterium*-mediated transformation in this process were proven. Silencing of the *HvCKX2* gene via *Agrobacterium*, as in the case of *HvCKX1*, determined organ-specific changes resulting in higher productivity of the modified lines.

## Results

### Transformation efficiency via biolistic and *Agrobacterium*-mediated method

In total, 9 putative transgenic plants were selected from 934 immature embryos of Golden Promise and Scarlett after biolistic transformation. Six of them were confirmed as transgenic, giving the transformation efficiency of 1.59% and 0.16% respectively (Table 1). The *Agrobacterium*-mediated transformation of 1036 explants of Golden Promise and 869 immature embryos of Scarlett resulted in obtaining 36 transgenic plants of the first cultivar with a mean transformation efficiency of 3.47% and only 1 plant of the second cultivar (0.12%). The transformation efficiency of Golden Promise after biolistic transformation was more than twice as low compared with the *Agrobacterium*-mediated transformation (1.59% and 3.46%, respectively). These data for the cultivar Scarlett were very low (0.16% and 0.12%) and the differences were not significant.

**Table 1:** **Number of explants, selected plants and lines, and transformation efficiency after biolistic (experiment 2) and *Agrobacterium*-mediated transformation (experiments 4–6) with the silencing cassette/vector and an empty (pMCG161) vector as a control**

**Biolistic transformation**							
**Cultivar**	**Number of experiment/cassette**	**Number of expl. calli plantlets rooted p. PCR+**					**Transformation efficiency (%)**
Golden Promise	2. CKX2 line	314	189	96	6	5	**1.59 (±1.24)**
Scarlett	2. CKX2 line	620	414	30	3	1	**0.16 (±0.59)**
***Agrobacterium*-mediated transformation**							
Golden	4. pMCG/CKX2	421	186	387	36	28	6.65
Promise	4. pMCG161	100	57	63	4	3	3.00
	5. pMCG/CKX2	75	68	106	5	4	5.33
	6. pMCG/CKX2	440	57	4	1	1	0.23
	Total	**1036**			**45**	**36**	**3.47 (±2.82)**
Scarlett	4. pMCG/CKX2	507	142	2	1	1	0.20
	6. pMCG/CKX2	237	52	0	0	0	0
	6. pMCG161	125	11	0	0	0	0
	Total	**869**			**1**	**1**	**0.12 (±0.12)**

The integration of silencing for the *HvCKX2* cassette and selection cassette containing *bar* under the control of Ubi1 intron promoter in T_0_ and T_1_ plants was tested by PCR with at least three pairs of specific primers. 78% of putative transgenic T_0_ plants and all the tested lines were proved to be transgenic (data not shown).

### The productivity of T_0_ plants decreases after biolistic silencing of *HvCKX2* and increases after *Agrobacterium*-mediated silencing

The morphology of silenced, *in vitro* plants (T_0_) was similar to the control plants, and independent of the method of transformation. All of them set seeds. The mean productivity of T_0_ plants, which is expressed as the number of grains and grain yield, the weight and CKX activity in the bulked samples of T_1_ roots of cv. Golden Promise and cv. Scarlett, transformed with a silencing cassette for the *HvCKX2* gene via the biolistic method, is presented in Table 2. Almost all the data were lower in transgenic compared to control *in vitro* plants in both cultivars. Control, non-silenced plants were obtained in the same conditions of *in vitro* culture. The mean number of grains in transgenic plants was 32% lower in Golden Promise and 51% lower (for one Scarlett plant) compared to the control; grain yield was 39% and 52% lower respectively. Similar lower data were obtained for 1000 grain weight and mean weight of T_1_ roots in transgenic plants of Golden Promise compared to the control. The data of CKX activity for bulked samples of T_1_ roots of Golden Promise were almost the same in the groups of transgenic and control plants and, for Scarlett, substantially higher in transgenic plants. There was no effect of silencing in the T_1_ roots of lines transformed via the biolistic method.

**Table 2:** **Mean productivity of T_0_ plants, the weight and the CKX activity in the roots of cv. Golden Promise and cv. Scarlett transformed with the silencing cassette for *HvCKX2 *gene via the biolistic method**

**Cultivar (number of plants)**	**Number of grains**	**Grain yield (g)**	**1000 grain weight (g)**	**Mean weight of T_1_ roots (mg)**	**Relative CKX activity in T_1_ roots**
**Golden Promise**					
Biolistic (5)	163.3 ±88.38	4.70 ±3.03	25.6 ±9.29	38.8 ±13.26	1.03 ±0.12
Control (3)	240.3 ±31.9	7.73 ±1.34	32.2 ±0.59	44.6 ±2.14	1.00 ±0.00
**Scarlett**					
Biolistic (1)	93	2.99	32.1	36.3	2.37
Control (6)	191.0 ±42.1	6.18 ±1.05	32.9 ±3.28	35.4 ±4.97	1.00 ±0.00

The mean productivity of T_0_ plants, the weight and CKX activity in the bulked samples of T_1_ roots of cv. Golden Promise transformed with a silencing cassette for the *HvCKX2* gene via the *Agrobacterium*-mediated method is presented in Table 3. Control, non-silenced plants were obtained in the same conditions of *in vitro* culture and selection. The data are from two different experiments, 4 and 5 (see Table 1), and in both of them the results were higher in the groups of plants transformed with a silencing cassette compared with the control plants. The mean number of grains per plant increased to 163% and 144% and the grain yield was 167% and 135% higher, depending on the experiment. The mean 1000 grain weight was similar for *Agrobacterium*-silenced and control lines, as was the mean weight of T_1_ roots, although in this case only for plants from experiment 4. Additionally, CKX activity in bulked samples of T_1_ roots, obtained after *Agrobacterium*-mediated silencing, in contrast to the data from the biolistic method, was decreased by over 29% compared to the non-silenced control.

**Table 3:** **Mean productivity of T_0_ plants, the weight and relative CKX activity in the roots of cv. Golden Promise transformed with a silencing cassette of *HvCKX2 *gene via the *Agrobacterium*-mediated method**

**Golden Promise (number of plants – no. of experiment)**	**Number of grains**	**Grain yield (g)**	**1000 grain weight (g)**	**Mean weight of T_1_ roots (mg)**	**Relative CKX activity in T_1_ roots**
*Agrobacterium*					
(28 – exp. 4) (4 –	**148.3**^**p**^ ±43,9	**5.25**^**p**^ ±1.65	35.7 ±7.75	41.3 ±11.44^8^	0.58 ±0.37
exp. 5)	131.0 ±40.7	4.25 ±1.9	33.5 ±14.5	**23.8**^**p**^ ±2.89	nt
Control (4 – exp.					
4)*	90.8 ±28.6	3.15 ±1.13	34.2 ±3.97	38.3 ±8.61	0.82 ±0.23

* - plants transformed with an empty (without silencing cassette) pMCG161 vector.

^8^ - tested for 8 lines.

nt - not tested; ^**p**^ – P<0,05.

### The level of *HvCKX2* silencing and the enzyme CKX activity in the T_1_ progeny differ depending on transformation method

**Figure 1:**
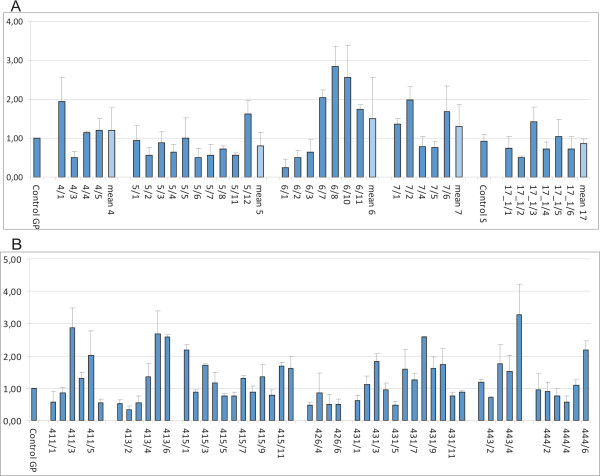
**A, B. Relative *HvCKX2 *transcript accumulation in 7 DAP spikes of T**_**1**_**plants after biolistic (A) and *Agrobacterium*-mediated transformation (B).**

Up to twelve progeny plants were examined from five biolistic-derived T_1_ lines and seven T_1_ lines obtained via *Agrobacterium*-mediated silencing. The data are related to the controls, assumed to be 1.00 (Table 4, Figure 1). Relative *HvCKX2* transcript accumulation in 7 DAP spikes of biolistic-silenced lines of Golden Promise ranged from 0.24 to 2.83, and for one line of Scarlett from 0.51 to 1.41 (Figure 1A, B). Some of the segregating progeny in each line showed significantly decreased relative *HvCKX2* transcript accumulation.

**Table 4:** **Relative CKX activity in developing and fully developed leaves and 7 DAP spikes in T_1_ lines of Golden Promise and one Scarlett line (S) silenced via biolistic and *Agrobacterium*-mediated methods**

**Biolistic method**						
	**Developing leaf**		**Developed leaf**		**Spike 7 DAP**	
Line*	**Range**	**Mean**	**Range**	**Mean**	**Range**	**Mean**
4	1.35 – 2.59	1.89 ±0.41	1.06 – 2.29	1.54 ±0.49	0.65 – 1.21	1.02 ±0.23
5**	0.78 – 4.84	2.55 ±1.57	1.01 – 14.11	3.44 ±3.83	0.40 – 1.13	0.75 ±0.28
6**	0.28 – 7.76	3.22 ±2.86	0.61 – 11.23	3.76 ±3.66	0.80 – 1.90	1.27 ±0.40
7	0.51 – 0.85	0.66 ±0.13	0.46 – 0.76	0.62 ±0.10	1.10 – 1.54	1.25 ±0.15
8	lack of germination					
17 S	0.55 – 1.78	1.01 ±0.44	0.12 – 1.28	0.89 ±0.49	0.58 – 1.13	0.70 ±0.22
***Agrobacterium*-mediated method**						
	**Developing leaf**		**Developed leaf**		**Spike 7 DAP**	
**Line**	**Range**	**Mean**	**Range**	**Mean**	**Range**	**Mean**
411	0.64 - 1.09	0.86 ±0.19	0.90 - 1.54	1.14 ±0.26	0.51 - 0.97	0.76 ±0.19
413	0.28 - 1.04	0.74 ±0.26	0.44 - 1.14	0.80 ±0.24	0.69 - 1.12	0.93 ±0.16
415**	0.39 – 1.13	0.70 ±0.25	0.40 – 1.19	0.76 ±0.27	0.40 – 1.33	0.90 ±0.28
426	0.23 – 1.05	0.60 ±0.34	0.34 – 0.97	0.61 ±0.25	0.58 – 1.13	0.87 ±0.23
431**	0.19 – 1.08	0.66 ±0.23	0.39 – 1.54	0.88 ±0.28	0.31 – 1.44	0.90 ±0.27
443	0.35 - 0.96	0.67 ±0.22	0.42 - 1.39	0.77 ±0.36	0.49 - 1.32	0.81 ±0.33
444	0.60 - 1.21	0.90 ±0.25	0.21 - 1.08	0.56 ±0.32	0.91 - 1.18	1.03 ±0.12

* – 5 to 6 plants tested in each line.

** – 10 to 12 plants tested.

S – Scarlett line.

Lack of significant differences (P<0,05).

Similar results of relative *HvCKX2* transcript accumulation in 7 DAP spikes were obtained in lines transformed with *Agrobacterium*; again the data for some of the progeny significantly exceeded the control level, 1.00 (Figure 1A). The range of data for seven lines was from 0.34 to 3.26. The *HvCKX2* transcript level in at least 1/3 of segregating progeny of lines 411, 413, 426, and 431 was reduced to about 50%.

The relative activity of CKX enzyme was measured in developing and fully developed leaves and 7 DAP spikes in T_1_ plants obtained via biolistic and *Agrobacterium*-mediated methods (Table 4). The range of the data for developing leaves of four biolistic-silenced Golden Promise lines were from 0.28 to 7.76 and for developed leaves from 0.46 to 14.11. These data in most of the progeny dramatically exceeded the relative activity in the control (1.00). The results of enzyme activity in spikes ranged from 0.40 to 1.90 and some segregated progeny in two of five lines, lines 5 and 17, showed significantly decreased CKX activity. The data in line 17 positively correlated with the lower enzyme activity in the leaves as well as with the lower level of transcript in 7 DAP spikes (Figure 1 B).

The range of relative CKX activity in the lines silenced via *Agrobacterium* was from 0.19 to 1.21 for developing leaves, from 0.21 to 1.54 for developed leaves, and from 0.31 to 1.44 for 7 DAP spikes. The ranges of data were not so wide and their standard deviation not so high as in T_1_ plants obtained via the biolistic method. The lowest data obtained in all the tested lines suggested an occurrence of segregating progeny with significantly decreased CKX activity in measured organs.

### The phenotypic effect of *HvCKX2* silencing mediated via biolistic and *Agrobacterium* transformation in T_1_ plants

The mean productivity, measured as the number of seeds per plant and grain yield, was decreased in four out of five biolistic-derived T_1_ lines (three derived from Golden Promise and one from Scarlett), compared with the control lines (Table 5). In the case of line 7 the difference for grain yield was statistically significant. The 1000 grain weight in lines 4, 7 and 17 was significantly reduced as well. The mean data for the weight of roots, height of plants and spike number and length were more variable between the silenced lines and the control and there was not any general tendency. Additionally, five plants from line 5 out of twelve tested in two experiments (5/1, 5/3, 5/5, 5/9, 5/10) did not set seeds, three plants from line 6 out of twelve (6/4, 6/9, 6/12) did not grow and one, 6/5, developed only one spike. Most of the seeds in this line did not germinate. All the seeds of line 8 refused to germinate.

**Table 5:** Mean productivity and selected phenotypic characteristics of four Golden Promise T_1_ lines (two experiments) and one Scarlett line, silenced via biolistic method

**Cultivar / line**	**Disturbed plants (plant number)**	**Number of seeds**	**Grain yield (g)**	**1000 grain weight (g)**	**Weight of roots (mg)**	**Height of plants (cm)**	**Spike length (cm)**	**Number of spikes setting seeds**
Golden Promise – experiment I								
5*	14 – 22 empty spikes (5/1, 5/3, 5/5)	101.3 ±29.3	2.61 ±0.56	26.2 ±2.74	90.7 ±19.0	63.3 ±3.14	8.5 ±0.57	11.5 ±7.58
6	no growth (6/4, 6/5) one spike (6/6)	112.8 ±74.6	3.68 ±2.56	32.2 ±5.65	62.6 ±16.5	58.8 ±6.83	9.13 ±0.42	6.80 ±3.70
Control GP I		123.5 ±47.3	4.28 ±1.24	36.85 ±7.61	52	71.28 ±2.80	8.75 ±5.02	9.03 ±4.80
Golden Promise – experiment II								
4	two spikes (4/6)	192 ±85.38	5.40 ±2.72	**26.6**^**p**^ ±5.62	51.83 ±9.64	83.33 ±3.56	8.22 ±0.66	13,2 ±9,0
5	15 - 22 empty spikes (5/9, 5/10)	172.50 ±103.78	5.85 ±3.76	33.48 ±1.77	56.00 ±9.01	76.50 ±3.02	9.22 ±0.44	13.7 ±5,5
6	no germin. (10 seeds); no growth (6/9, 6/12)	318.25 ±117.84	12.02 ±4.34	37.85 ±2.33	44.20 ±12.21	82.00 ±1.41	8.45 ±0.70	12.00 ±3.2
7		117.33 ±57.84	**3.46**^**p**^ ±1.99	**29.6**^**p**^ ±9.06	56.67 ±16.12	77.00 ±5.33	8.82 ±1.18	11.50 ±4.2
Control GP II		218 ±66.5	7.85 ±2.40	36.0 ±1.97	52.7 ±16.6	80.2 ±4,15	9.09 ±0.57	12.1 ±2.32
Scarlett								
17		126.3 ±81.4	3.73 ±2.58	**27.3**^**p**^ ±6.96	**103.0**^**p**^ ±14.8	58.8 ±11.4	7.63 ±1.41	8.17 ±3.31
Control S (3)		140.0 ±24.5	5.78 ±0.96	41.4 ±0.52	58.7 ±30,0	70.0 ±3.00	8.60 ±0.70	9.33 ±3.21

* – 4 to 6 plants in each line; ^**p**^ – P<0,05.

**Figure 2:**
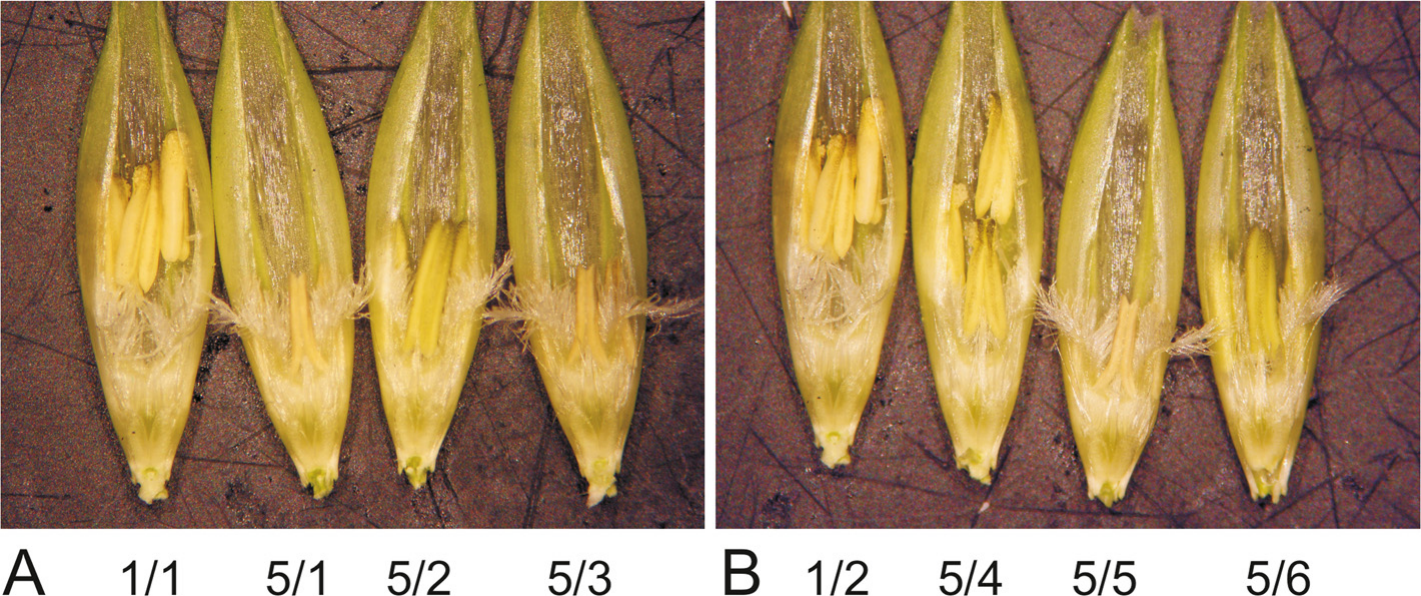
**Spikelets with the anthers in control 1/1 plant (first left; A, B) following biolistic-silenced 5/1, 5/2, 5/3 T**_**1**_**plants (A) and in control 1/2 plant following 5/4, 5/5, 5/6 T**_**1**_**plants (B). 5/1 – 5/6 plants are the progeny from T**_**0**_**5 plant**.

As documented in Figure 2, T_1_ plants 5/1, 5/3 and 5/5, which showed a lack of setting seeds, did not develop functional anthers. This phenotype was observed again in 5/9 and 5/10 plants of the same line in the second experiment (Table 5). The size of the anthers was considerably reduced, and there was no pollen production. The relative level of *HvCKX2* transcript in the 7 DAP spikes (Figure 2) as well as developing and developed leaves of these plants (not shown) was around 1.00, similar to the control plants. However, CKX activity in young leaves of the same plants ranged from 2.01 to 4.84, and for developed leaves from 1.01 to 4.01. These data of enzyme activity were in the range of data for seed-setting progeny from lines 5 and 6 of Golden Promise lines (see Table 4). CKX activity in the leaves of these plants was unbalanced and exceeded the level of the control plants. Other data were obtained for CKX activity in 7 DAP spikes. The range for all T_1_ plants of line 5 was from 0.40 to 1.13 (Table 4). The enzyme activity for individual, non-seed-setting plants of this line were: 0.48 for plant 5/1, 0.40 for plant 5/3, 0.46 for plant 5/5, 0.56 for plant 5/9 and 0.40 for plant 5/10. None of the other seed-setting plants from lines 5 and 6 had such low activity of CKX in 7 DAP spikes (the range was from 0.77 to 1.90).

There was no problem with setting seeds in all tested 47 T_1_ plants derived from 7 lines silenced via *Agrobacterium* and 28 T_1_ plants, progeny of 4 control lines, or in any other of the thousands obtained via *Agrobacterium* and tested in our laboratory. The mean productivity expressed as the number of grains and grain yield was higher in silenced (via *Agrobacterium*) lines compared with the control lines in both experiments and in all individual lines (Table 6). The mean 1000 grain yield exceeded the mean control value in four lines. The mean height of 6 out of 7 tested silenced lines was higher from 1.0 cm to 4.5 cm than the mean for the four control lines, which was 60.0 cm (±2.6) and for line 444 the difference was statistically significant. The mean spike length in the *Agrobacterium*-silenced group was only slightly higher, and the number of spikes was from 0.47 to 2.3 higher in 6 out of 7 tested lines compared with the control. This means that silenced T_1_ plants developed from 0.5 to 2 spikes per plant more than non-silenced *Agrobacterium*-transformed control T_1_ plants.

**Table 6:** **Mean productivity and selected phenotypic data of T_1_ control and silenced lines of Golden Promise obtained via *Agrobacterium*-mediated methods in two experiments**

**Cultivar / T1 line**	**Number of seeds**	**Grain yield (g)**	**1000 grain weight (g)**	**Weight of roots (mg)**	**Height of plants (cm)**	**Spike length (cm)**	**Number of spikes**
*Agrobacterium*-silenced – experiment I							
411*	123.2 ±67.5	3.63 ±1.9	29.9 ±2.9	50.7 ±8.1	57.3 ±2.7	8.15 ±0.74	6.83 ±2.4
413	121.3 ±49.8	4.04 ±1.4	34.1 ±3.6	66.2 ±13.9	62.2 ±3.3	8.88 ±0.13	6.33 ±1.5
415	118.7 ±15.9	3.62 ±0.7	30.4 ±4.9	105.8 ±38.9	63.4 ±3.7	8.50 ±0.55	6.67 ±0.7
426	120.8 ±24.4	3.95 ±0.8	32.8 ±2.0	142.7 ±91.2	63.6 ±1.5	8.78 ±0.22	6.40 ±1.0
431	110.0 ±20.5	4.14 ±0.8	37.6 ±2.3	97.8 ±44.8	63.0 ±1.4	7.73 ±0.63	7.00 ±1.2
443	125.5 ±22.9	3.75 ±0.9	29.7 ±2.2	162.8 ±50.2	61.0 ±2.0	8.00 ±0.52	7.83 ±0.7
444	103.5 ±29.3	3.53 ±1.2	34.1 ±4.1	108.8 ±29.6	**64.5**^**p**^ ±3.4	8.48 ±0.58	5.83 ±1.3
*Agro*-silenced							
mean	117.5 ±35.4	3.80 ±1.11	32.7 ±4.1	105.0 ±57.0	62.1 ±3.38	8.33 ±0.63	6.71 ±1.5
Control – experiment I							
405	96.8 ±19.1	3.00 ±0.6	32.7 ±13.4	57.0 ±23.2	57.5 ±2.1	7.58 ±1.04	5.50 ±1.3
406	111.7 ±33.3	3.35 ±0.7	30.5 ±3.5	109.5 ±68.6	60.5 ±2.9	8.34 ±1.08	6.50 ±1.4
407	99.3 ±19.3	3.00 ±0.6	30.3 ±3.6	141.7 ±26.8	60.0 ±2.8	8.37 ±0.92	5.83 ±1.2
408	100.3 ±26.4	3.28 ±0.7	32.9 ±2.0	79.0 ±24.3	61.0 ±1.5	8.57 ±0.60	5.67 ±1.1
Control mean	102.5 ±24.6	3.17 ±0.7	31.5 ±5.8	100.4 ±78.5	60.0 ±2.6	8.27 ±0.91	5.86 ±1.2
Experiment II							
415	268.2 ±74.8	11.12 ±3.2	41.8 ±7.7	53.8 ±5.4	**73.8**^**p**^ ±2.6	8.80 ±0.56	15.2 ±3.7
431	294.3 ±86.1	10.82 ±3.4	36.6 ±1.6	54.2 ±12.7	79.8 ±4.0	9.37 ±0.79	15.5 ±3.3
*Agro*-silenced mean	281.2 ±78.1	10.98 ±3.16	39.2 ±5.9	54.0 ±9.30	76.6 ±4.41	9.09 ±0.72	15.3 ± 3.3
Control	269.3 ±68.3	10.44 ±2.3	39.0 ±2.2	55.2 ±4.4	80.7 ±4.0	9.20 ±0.53	13.2 ±2.8

* – 5 to 6 plants in each line, ^**p**^ – P<0.05.

## Discussion

Based on RNAi processes, gene silencing has already been proven to be an efficient approach for functional genomics in cereals [1,4,24,25]. The stability of silencing cassette expression as well as directed gene silencing is one of the key requirements for the successful application of transgenic lines in basic research of gene function and agricultural genetic improvement. In this study we applied the posttranscriptional gene silencing process (PTGS) to silenced *HvCKX2* in two barley cultivars by two different transformation methods: biolistic and *Agrobacterium*.

Golden Promise cultivar is widely used in barley biotechnology/functional genomics, because of its susceptibility to *Agrobacterium*-mediated transformation, which was also confirmed in this paper. The second one, cv. Scarlett, showed very low transformation ability. The data obtained for Golden Promise with the *bar* selection system were 3.5% for *Agrobacterium*-mediated transformation and less than half this value, 1.6%, after biolistic transformation. A comparable result for the same cultivar of barley, doubled transformation efficiency with *Agrobacterium* compared to particle bombardment, was obtained by Travella et al. [22]. These results are also in agreement with those obtained for rice [21,26]. The second cultivar tested, expressing very low efficiency after *Agrobacterium*-mediated transformation, also showed very low efficiency after the biolistic method. We might assume that the susceptibility to bacteria was not the only limiting factor for cv. Scarlett transformation. This result is against the hypothesis that genotype dependence of susceptibility to *Agrobacterium* might be the limiting factor in applying the *Agrobacterium*-mediated method, which should not appear in the biolistic method [27].

The silencing of *HvCKX2* by the biolistic method determined low productivity and by *Agrobacterium* high productivity of T_0_ plants. This general tendency of plant productivity was also transmitted to the next generation. Higher productivity was the result of a higher number of seeds and grain yield, higher 1000 grain weight as well as increased (by 7.5%) height of plants and higher (from 0.5 to 2.3) numbers of spikes. We also documented that this higher productivity was correlated with lower levels of *HvCKX2* transcript in 7 DAP spikes and decreased CKX activity in leaves and 7 DAP spikes in the progeny of lines silenced via *Agrobacterium*. The tissues/organs appropriate for analysis were chosen based on temporal and spatial expression of *HvCKX2* measured in developing wild barley cultivars of Golden Promise and Scarlett. These data were highest in 14 DAP spikes, followed by 7 DAP and 0 DAP, as well as in the leaves (not yet published). The phenotypic result of silencing of *HvCKX2* in these tissues via *Agrobacterium* was higher productivity and increased height of silenced lines. These results confirm once again the hypothesis that spatial and temporal differences in expression contributed to functional differentiation [1,4]. The higher productivity might be the result of lower *HvCKX2* transcript in developing spikes and decreased CKX activity. Increased height of silenced plants may be dependent on decreased CKX activity in the leaves, estimated at a high level in the wild plants. Similar results of *HvCKX1* silencing via *Agrobacterium* in Golden Promise, which correlated with the specific organs, were observed in our previous research [1]. The highest expression of the gene in wild-type plants was in 7 DAP spikes, followed by 14 DAP and 0 DAP, as well as in the roots. In that experiment, the silencing of *HvCKX1* led to higher plant productivity as well as higher mass of the roots, although the height of the plants was reduced. The reduced expression of another *CKX* gene in rice, *OsCKX2*, caused cytokinin accumulation in the inflorescence meristems, increased the number of reproductive organs, and increased grain number and yield [4]. It was also documented that halophyte variants of *TaCKX6D1*, a wheat ortholog of rice *OsCKX2*, was associated with grain weight in hexaploid wheat [28]. Newly published research on phylogenic and sequence analysis showed that *CKX1* and *CKX2* are closely related in clade Ia of Poaceae and are physically linked [6]. The authors hypothesized that both genes might have similar functions, which is supported by our earlier research on *HvCKX1*[1] and *HvCKX2* in this paper.

The opposite result of the plant productivity obtained in T_0_ and T_1_ lines, when *HvCKX2* was silenced with the biolistic method, might be explained by somaclonal variation [29]. This term, describing the phenotypic variability among plants of *in vitro* origin, includes genetic and epigenetic modifications [18]. Both types of modifications in biolistic-derived plants are caused by a physically destructive method and integration of many, mostly rearranged copies of a transgene, the result of which is frequently determined transgene silencing [22]. Due to resource limitation, the copy number was not examined in this study. However based on the knowledge from previously published papers [16,21,22,30] the phenotypic differences in lines generated by two transformation methods might be attributed to different copy numbers of transgene integrated to the genome as well as the DNA/transgene rearrangements. In such situations our silencing cassette introduced by the biolistic method might disturb the effect of silencing and the whole phenotype, otherwise visible in the group of *Agrobacterium*-silenced plants. This effect, depending on the method of transformation, might be especially distinct in the case of the silencing of developmentally regulated genes, like *CKX*s. Besides lower plant productivity, it also caused a lack of germination in one line and inability of seed setting in half of the progeny of another line (out of five tested). The primary reason was a lack of functional anthers. This was correlated with the lowered to 50% - 60% CKX activity in 7 DAP spikes and it was not observed in seed-setting plants of biolistic origin. However, lowered CKX activity has also been proven in *Agrobacterium*-silenced lines, where it resulted in higher productivity. The explanation of these differences might be observed, unbalanced CKX activity in the whole biolistic-derived plants – very high in the leaves and very low in the 7 DAP spikes. This result proved the earlier reported observations in other plant species that *CKX*s respond differently to various stresses [31,32]. The effect of *SBEIIa* silencing on starch metabolism in durum wheat lines obtained with the two methods of transformation, biolistic and *Agrobacterium*, was genotype and protocol independent [33]. However, these results are not directly comparable with ours, because in that report two different cultivars were transformed with one of two methods, the silenced genes influenced only starch metabolism (causing alterations in granule morphology and starch composition, leading to high amylose wheat), and there was a lack of detailed data on productivity, possibly because of the character of the genes tested.

The final effect of the silencing observed in biolistic-derived and *Agrobacterium*-derived plants was also different. The transcript level in segregating progeny of lines transformed by both methods was similar, decreased to 24% (biolistic) and to 34% (*Agrobacterium*), and it was reduced in about 1/3 of plants by more than 50%. The consequence of this reduction of transcript in *Agrobacterium*-derived plants was decreased CKX activity in developing and developed leaves as well as in 7 DAP spikes. Otherwise the enzyme activity for developing and developed leaves of the lines of biolistic origin, especially in cv. Golden Promise, was very high, exceeding the relative level for control lines. This imbalanced effect of the low level of the transcript and very high CKX activity suggest disturbances of developmental processes, which are naturally guided by small RNA at the transcriptional (DNA) and posttranscriptional (RNA) levels [34,35]. Both PTGS and TGS may be influenced by environmental and developmental factors [20]. Such disturbances in *HvCKX2* experimentally silenced by *Agrobacterium* have not occurred, proving the applicability of the method for gene silencing of developmentally regulated genes.

## Conclusions

The results of RNAi–mediated *HvCKX2* silencing in T_0_ plants and T_1_ lines obtained by two different methods were contrasting. *Agrobacterium*-silenced lines showed expected lower levels of transcript in the 7 DAP kernels and decreased CKX enzyme activity in the leaves and 7 DAP kernels. The phenotypic effect was higher plant productivity expressed by a higher number of seeds and grain yield as well as plant height. Contrasting data were obtained in lines with *HvCKX2* silenced by the biolistic method. The first effect of silencing, which was a lower transcript level in 7 DAP of segregating progeny, was comparable with that in *Agrobacterium*-silenced lines. However, a decrease of CKX activity in the spikes determined the lack of pollen development and not seed-setting phenotype. Additionally, the activity of the enzyme in the leaves of all Golden Promise progeny was imbalanced, exceeding the level of the control. The final phenotypic effect was a decrease of plant productivity. We suggest that the differences in silencing effect observed in transgenic lines generated by two transformation methods might be the result of different patterns of transgene integration including copy number and/or transgene rearrangements. The limitations of both transformation methods are discussed.

Presented results prove the applicability of *Agrobacterium*-mediated silencing and inapplicability of biolistic silencing for developmentally regulated genes.

## Methods

### Vector construction

**Figure 3:**
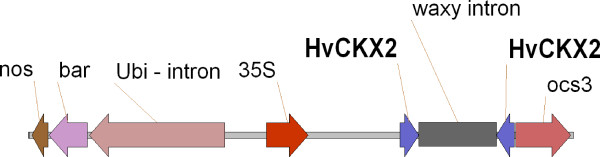
**The schematic structure of Ubi-intron/*bar *selection cassette and silencing cassette for silencing of *HvCKX2 *gene via *Agrobacterium *and biolistic method**.

The hpRNA type of silencing cassette was constructed in the pMCG161 (http://www.chromdb.org/mcg161.html) binary vector. The T-DNA of the vector contained the *bar* selection gene under the control of the Ubi1 intron promoter and two restriction sites for cloning the RNAi construct, separated by a rice waxy intron and driven by the cauliflower mosaic virus 35S promoter. A 259 bp fragment of the *HvCKX2* gene (NCBI accession AF540382.1), which is conserved amongst the *CKX* gene family, was cloned into the pDRIVE vector (Qiagen) and amplified with primers containing restriction sites for cloning: SpeI and SacI, and RsrII and AvrII. The sequences of the primers were: CKX2s 5^′^-TTCGGACCGACTAGTGAGGCGAACTCTGGATA-3^′^ and CKX2a 5^′^-TTCCTAGGGAGCTCAAACTGACCCAGACCACCAAGA-3^′^. After restriction and cleaning, the fragments were ligated to the silencing cassette in the sense and antisense orientation (Figure 3). The resulting vector, pMCG/*HvCKX2*, was electroporated to the DH5α strain of *Escherichia coli*. Single colonies were selected on an LB medium containing 35 mg l^-1^ chloramphenicol. After confirmation of correct cloning by restriction analysis with a series of enzymes, the vector was electroporated to *Agrobacterium tumefaciens*, AGL1 strain.

### Plant material, *Agrobacterium*-mediated transformation and *in vitro* culture

The silencing cassette was introduced into two barley cultivars: Scarlett and Golden Promise, via *Agrobacterium* transformation. Immature embryos were isolated and *in vitro* cultured according to the protocols described by Przetakiewicz et al. [36] and Zalewski et al. [1]. 2 mg l^-1^ phosphinothricin was used for selection during the whole period of post-transformation culture. The culture of the bacterial strain AGL1 and the transformation of barley with pMCG/*HvCKX2* were performed as previously described [37].

### Biolistic transformation

Immature embryos of Golden Promise and Scarlett plants were transformed by particle-bombardment using a PDS 1000 He device (BioRad) with 1100 psi rupture discs. After isolation from adult plants, embryos were pre-cultured in the dark for 24 h and then were transferred to an osmotic medium containing 0.3 M mannitol for 4 h before bombardment and were cultured on this medium for the next 16 h in the dark. Protocol and media for the transformation method were previously described by Harwood et al. [38]. A line fragment containing selection and silencing cassettes cut with *Hind*III and *Not*I from pMCG/HvCKX2 vector was used for transformation. Gold microprojectiles of 1 μm diameter were suspended in 5 μl of DNA solution (1 μg/μl). 20 μl of 0.1 M spermidine and 50 μl of 2.5 M CaCl_2_ were mixed with particles coated with DNA and then centrifuged for 1 min. Supernatant was removed, and after two further washes with 99.8% ethanol, 3.5 μl of suspension was used for one shot. The conditions and media used for *in vitro* culture and selection (2 mg l^-1^ phosphinothricin) of biolistic-transformed embryos was the same as in the case of *Agrobacterium* transformation.

### PCR analysis of T_0_ and T_1_ plants

Genomic DNA was isolated from the young leaves using a modified version of the CTAB method [39]. Polymerase chain reaction was performed in a 25 μl reaction mixture containing 150 ng of template genomic DNA, 100 μM of each dNTP, 3 μM of each primer, 1 U of DNA polymerase (Invitrogen), 2 mM MgCl_2_, and 1x DNA polymerase buffer. PCR analysis was performed with six pairs of primers. Three of them produced fragments of various lengths from sense and antisense inserts: a 703 bp fragment amplified with pM1,2; a 624 bp fragment with pM3,4; and a 1103 bp fragment with pM5,6 primers on pMCG/*HvCKX2*. Three pairs of qOCS oligos were used for both standard and quantitative PCR. The qOCS oligos primed the amplification of the OCS 3′ terminator fragments of 171 bp (qOCS1,2), 171 bp (qOCS3,4), and 182 bp (qOCS5,6). The sequences of the primers and PCR amplification conditions were previously reported by Zalewski et al. [1]. Each plant was tested with at least 3 different pairs of primers. Transformation efficiency was estimated as the percentage of initial explants giving rise to PCR positive plants. T_1_ lines tested in the experiments were from independent transformation events.

### Quantitative RT-PCR

The total RNA was isolated from developing leaves and immature kernels 7 and 14 days after pollination (DAP). For leaves a TRI Reagent kit (Applied Biosystems) was applied according to the manufacturer’s protocol. The total RNA from the kernels was isolated using a modified version of the TRI Reagent protocol with SDS extraction [40]. Isolated RNA was treated using DNase I Recombinant, RNase-Free (Roche) to minimize the bias in PCR data caused by traces of genomic DNA contamination. The cDNA was synthesized from 500 ng of RNA according to the manufacturer’s instructions using a RevertAid First Strand cDNA Synthesis Kit (Fermentas).

For a quantitative analysis of transcript accumulation of the silenced gene, three pairs of primers were designed: qAct1.2 for the beta-actin gene as a reference, qCKX21.2 and qHC2_3,4 for *HvCKX2* as a target gene. The sequences of primers for amplification of the 133 bp cDNA of the reference gene were qAct1, 5^′^-AGCAACTGGGATGACATGGAG-3^′^ and qAct2, 5^′^-GGGTCATCTTCTCTCTGTTGGC-3^′^. The sequences of primers for cDNA of the *HvCKX2* amplification wereqCKX21 5^′^-CGCGGAACTCTGGATAAATGTCTTG-3^′^ and qCKX22 5^′^-AGTTCTGTTCTGGTGAGCAAGTGAC-3^′^; qHC2_3 5^′^- CCATATTGCTCTACCCAGTGAAG-3^′^ and qHC2_4 5^′^-GGTTCTGTGGTGTAGTTTGGAAG-3^′^. Amplification products were 217 bp and 187 bp long, respectively.

The real-time PCR cycling conditions were: 45 cycles with an initial denaturation at 95°C for 10 min, denaturation at 95°C for 30 s, annealing at 60°C for 30 s, and a melting curve at 70–95°C (5 s per step). The relative expression level of the *HvCKX2* gene was calculated according to the ΔΔCt method using the beta-actin gene as a normalizer. The values of three replicates of each sample were used for the calculation. The control lines transformed with the empty vector pMCG161 (*Agrobacterium*) or non-transgenic *in vitro* plants (biolistic) were designed as calibrator samples with their expression values set to 1.00 (control = 1.00).

### Analysis of cytokinin dehydrogenase activity

Activity of the enzyme was tested in young, developing and fully developed leaves, 7 DAP spikes and roots from 5-day seedlings. The plant material was powdered with liquid nitrogen using a hand mortar, and extracted with a 2-fold excess (v/w) of 0.2 M Tris–HCl buffer, pH 8.0, containing 1 mM phenylmethylsulfonyl fluoride (PMSF) and 0.3% Triton X-100. Assay was performed according to Frebort et al. [41]. Plant samples were incubated in a reaction mixture consisting of 100 mM McIlvaine buffer, 0.25 mM of the electron acceptor dichlorophenolindophenol, and 0.1 mM of substrate (N6-isopentenyl adenine). The volume of the enzyme sample used for the assay was adjusted based on the enzyme activity. The incubation temperature was 37°C for 1–16 h. After incubation the reaction was stopped by adding 0.3 ml of 40% trichloroacetic acid (TCA) acid and 0.2 ml of 4-aminophenol (2% solution in 6% TCA). The product concentration was determined by scanning the absorption spectrum from 300 nm to 700 nm. The protein concentration was assayed according to the Bradford method [42] with bovine serum albumin as the standard. The measurements of activity were done in triplicate. To calculate relative values, control lines (as above) were designed as a calibrator with the enzyme activity values set to 1.00.

### Analysis of phenotypic data

1000 grain weight was calculated by dividing grain yield from each plant by the total number of seeds and multiplying by 1000. Mass of the roots was estimated in 5-day old seedlings, germinated in Petri dishes on wet blotting paper. The roots from each plant were cut 3 mm from the base, dried on blotting paper and weighed. After that they were powdered with liquid nitrogen to perform further analysis and the seedlings continued their growth to develop new roots for four more days. At least 5 to 6 PCR positive plants were tested in each line.

Standard deviations were calculated using the Microsoft Excel 2003 program.

Statistical analysis was done using the Statistica (StatSoft) program. An analysis of variance for normality was calculated using the Shapiro-Wilk test. Homogeneity of variance was verified by Brown-Forsythe test. Tukey’s honest significance test for the single-step multiple comparison procedure was used to determine which means were significantly different at the level P < 0.05 from one another.

## Competing interests

The authors declare that they have no competing interests.

## Authors' contributions

WZ carried out most of the experiments: vector construction, *Agrobacterium*-mediated and biolistic transformation, selection and analysis of plants; WO coordinated vector construction and took part in discussion of the project; SG participated in vector construction and analysis; A N-O coordinated the work and wrote the manuscript. All authors read and approved the final manuscript.

## References

[B1] Zalewski W Galuszka P Gasparis S Orczyk W Nadolska-Orczyk A Silencing of the *HvCKX1* gene decreases the cytokinin oxidase/dehydrogenase level in barley and leads to higher plant productivity J Exp Bot 2010 61 1839 1851 20335409 10.1093/jxb/erq052

[B2] Werner T Motyka V Laucou V Smets R Van Onckelen H Schmulling T Cytokinin-deficient transgenic *Arabidopsis* plants show multiple developmental alterations indicating opposite functions of cytokinins in the regulation of shoot and root meristem activity Plant Cell 2003 15 1 20 14555694 10.1105/tpc.014928 PMC280559

[B3] Schmulling T Werner T Riefler M Krupkova E Bartrina Y Manns I Structure and function of cytokinin oxidase/dehydrogenase genes of maize, rice, *Arabidopsis* and other species J Plant Res 2003 116 241 252 12721786 10.1007/s10265-003-0096-4

[B4] Ashikari M Sakakibara H Lin S Yamamoto T Takashi T Nishimura A Angeles ER Quian Q Kitano H Matsuoka M Cytokinin oxidase regulates rice grain production Science 2005 309 741 745 15976269 10.1126/science.1113373

[B5] Galuszka P Frebortova J Werner T Mamoru Y Strand M Schmulling T Frebort I Cytokinin oxidase/dehydrogenase genes in barley and wheat cloning and heterologous expression J Biochem 2004 271 3990 4002 10.1111/j.1432-1033.2004.04334.x 15479228

[B6] Mameaux S Cockram J Theil T Steuernagel B Stein N Taudien S Jack P Werner P Gray JC Greenland AJ Powell W Molecular, phylogenetic and comparative genomic analysis of the cytokinin oxidase/dehydrogenase gene family in the Poaceae Plant Biotechnol J10.1111/j.1467-7652.2011.00654.x 21838715

[B7] Schulte D Close TJ Graner A Langridge P Matsumoto T Muehlbauer G Sato K Schulman AH Waugh R Wise RP Stein N The international barley sequencing consortium – at the threshold of efficient access to the barley genome Plant Physiol 2009 149 142 147 19126706 10.1104/pp.108.128967 PMC2613708

[B8] Saisho D Takeda K Barley: emergence as a new research material of crop science Plant Cell Physiol 2011 52 724 727 21565909 10.1093/pcp/pcr049

[B9] Chuang CF Meyerowitz EM Specific and heritable genetic interference by double-stranded RNA in *Arabidopsis thaliana* Proc Nat Acad Sci 2000 9 4985 4990 10781109 10.1073/pnas.060034297 PMC18344

[B10] Wesley SV Helliwell CA Smith NA Wang M Rouse DT Liu Q Gooding PS Singh SP Abbott D Stoutjesdijk PA Robinson SP Gleave AP Green AG Waterhouse PM Construct design for efficient, effective and high-throughput gene silencing in plants Plant J 2001 27 581 590 11576441 10.1046/j.1365-313x.2001.01105.x

[B11] Watson JM Fusario AF Wang M Waterhouse PM RNA silencing platforms in plants FEBS Lett 2005 578 5982 5987 16139270 10.1016/j.febslet.2005.08.014

[B12] Miki D Itoh R Shimamoto K RNA silencing of single and multiple members in a gene family of rice Plant Physiol 2005 138 1903 1913 16172097 10.1104/pp.105.063933 PMC1183382

[B13] Fu D Uauy C Blechl A Dubcovsky J RNA interference for wheat functional gene analysis Transgenic Res 2007 16 689 701 17952622 10.1007/s11248-007-9150-7

[B14] Gubler F Hughes T Waterhouse P Jacobsen J Regulation of dormancy in barley by blue light and after-ripening: effects on abscisic acid and gibberellin metabolism Plant Physiol 2008 147 886 896 18408047 10.1104/pp.107.115469 PMC2409010

[B15] Gasparis S Orczyk W Zalewski W Nadolska-Orczyk A The RNA-mediated silencing of one of the Pin genes in allohexaploid wheat simultaneously decreases the expression of the other, and increases grain hardness J Exp Bot 2011 62 4025 4036 21504879 10.1093/jxb/err103

[B16] Rao AQ Bakhsh A Kiani S Shahzad K Shahid AA Husnain T Riazuddin S The myth of plant transformation Biotechnol Adv 2009 27 753 763 19508888 10.1016/j.biotechadv.2009.04.028

[B17] Nadolska-Orczyk A Orczyk W Przetakiewicz A *Agrobacterium*-mediated transformation of cereals – from technique development to its application Acta Physiol Plant 2000 22 77 88

[B18] Miguel C Marum L An epigenetic view of plant cells cultured in vitro: somaclonal variation and beyond J Exp Bot 2011 62 3713 3725 21617249 10.1093/jxb/err155

[B19] Krizova K Fojtova M Depicker A Kovarik A Cell culture-induced gradual and frequent epigenetic reprogramming of invertedly repeated tobacco transgene epialleles Plant Physiol 2009 149 1493 1504 19129419 10.1104/pp.108.133165 PMC2649402

[B20] De Neve M De Buck S De Wilde C Van Houdt H Strobbe I Jacobs A Van Montagu M Depicker A Gene silencing results in instability of antibody production in transgenic plants Mol Gen Genet 1999 260 582 592 9928938 10.1007/s004380050932

[B21] Dai S Zheng P Marmey P Zhang S Tian W Chen S Beachy RN Fauquet C Comparative analysis of transgenic rice plants obtained by Agrobacterium-mediated transformation and particle bombardment Mol Breeding 2001 7 25 33

[B22] Travella S Ross SM Harden J Everett C Snape JW Harwood WA A comparison of transgenic barley lines produced by particle bombardment and *Agrobacterium*-mediated techniques Plant Cell Rep 2005 23 780 789 15761662 10.1007/s00299-004-0892-x

[B23] Barlett JG Alves SC Smadley M Snape AW Harwood WA High-throughput Agrobacterium-mediated barley transformation Plant Methods 2008 4 22 18822125 10.1186/1746-4811-4-22 PMC2562381

[B24] Yan L Loukoianov A Blechl A Tranquilli G Ramakhrisna W SanMiquel P Bennetzen JL Echenique V Dubcovsky J The wheat *VRN2* gene is a flowering repressor down-regulated by vernalization Science 2004 303 1640 1644 15016992 10.1126/science.1094305 PMC4737501

[B25] Travella S Klimm TE Keller B RNA interference-based gene silencing as an efficient tool for functional genomics in hexaploid bread wheat Plant Physiol 2006 141 6 20 16861570 10.1104/pp.106.084517 PMC1557595

[B26] Khanna HK Raina SK Elite Indica transgenic rice plants expressing modified Cry1Ac endotoxin of *Bacillus* thuringiensis show enhanced resistance to yellow stem borer (*Sciropophaga incertulas*) Transgenic Res 2002 11 411 423 12212843 10.1023/a:1016378606189

[B27] Altpeter F Baisakh N Beachy R Bock R Capell T Christou P Daniell H Datta K Dix PJ Fauquet C Huang N Kohli A Mooibroek H Nicholson L Nguyen G Raemakers K Romano A Somers DA Stoger E Taylor N Visser R Particle bombardment and the genetic enhancement of crops: Myths and realities Mol Breeding 2005 15 305 327

[B28] Zhang L Zhao Y-L Gao L-F Zhao G-Y Zhou R-H Zhang B-S Jia J-Z TaCKX6-D1, the ortholog of rice OsCKX2, is associated with grain weight in hexaploid wheat New Phytol 2012 195 574 584 22670578 10.1111/j.1469-8137.2012.04194.x

[B29] Larkin PJ Scowcroft WR Somaclonal variation – a novel source of variability from cell cultures for plant improvement Theor Appl Gen 1981 60 197 214 10.1007/BF02342540 24276737

[B30] Brugiere N Jiao S Hantke S Zinselmaier C Roessler JA Niu X Jones RJ Habben JE Cytokinin oxidase gene expression in maize is localized to the vasculature, and is induced by cytokinins, abscisic acid, and abiotic stress Plant Physiol 2003 132 1228 1240 12857805 10.1104/pp.102.017707 PMC167063

[B31] Kohli A Leech M Vain P Laurie DA Christou P Transgene organization in rice engineered through direct DNA transfer supports a two-phase integration mechanism mediated by the establishment of integration hot spots Proc Natl Acad Sci USA 1998 95 7203 7208 9618563 10.1073/pnas.95.12.7203 PMC22782

[B32] Vyroubalova S Vaclavikova K Tureckova V Novak O Smehilova M Hluska T Ohnoutkovw L Frebort I Galuszka P Characterization of new maize genes putatively involved in CK metabolism and their expression during osmotic stress in relation with cytokinin levels Plant Physiol 2009 151 433 447 19641027 10.1104/pp.109.142489 PMC2735981

[B33] Sestili F Janni M Doherty A Botticella E D’Ovidio R Masci S Jones HD Lafiandra D Increasing the amylase content of durum wheat through silencing of the SBEIIa genes BMC Plant Biol 2010 10 144 20626919 10.1186/1471-2229-10-144 PMC3095290

[B34] Vaucheret H Fagart M Transcriptional gene silencing in plants: targets, inducers and regulators Trends Genet 2001 17 29 35 11163919 10.1016/s0168-9525(00)02166-1

[B35] Chen X Small RNAs and their roles in plant development Ann Rev Cell Dev Biol 2009 25 21 44 19575669 10.1146/annurev.cellbio.042308.113417 PMC5135726

[B36] Przetakiewicz A Orczyk W Nadolska-Orczyk A The effect of auxin on plant regeneration of wheat, barley and triticale Plant Cell, Tissue Organ Cul 2003 73 245 256

[B37] Przetakiewicz A Karas A Orczyk W Nadolska-Orczyk A Agrobacterium-mediated transformation of polyploid cereals. The efficiency of selection and transgene expression in wheat Cell Mol Biol Lett 2004 9 903 917 15647806

[B38] Harwood WA Ross SM Cilento P Snape JW The effect of DNA/gold particle preparation technique, and particle bombardment device, on the transformation of barley (*Hordeum vulgare*) Euphytica 2000 111 67 76

[B39] Murray AA Thompson WF Rapid isolation of high molecular weight plant DNA Nucl Acid Res 1980 8 4321 4325 10.1093/nar/8.19.4321 PMC324241 7433111

[B40] Prescott A Martin C A rapid method for the quantitative assessment of levels of specific mRNAs in plants Plant Mol Biol Rep 1987 4 219 224

[B41] Frebort I Sebela M Galuszka P Werner T Schmulling T Pec P Cytokinin oxidase/dehydrogenase assay: optimized procedures and applications Anal Bioch 2002 306 1 7 10.1006/abio.2002.5670 12069407

[B42] Bradford MM A rapid and sensitive method for the quantitation of microgram quantities of protein utilizing the principle of protein-dye binding Anal Bioch 1976 72 248 254 10.1016/0003-2697(76)90527-3 942051

